# Assessing the potential impact of COVID-19 on life expectancy

**DOI:** 10.1371/journal.pone.0238678

**Published:** 2020-09-17

**Authors:** Guillaume Marois, Raya Muttarak, Sergei Scherbov

**Affiliations:** 1 Asian Demographic Research Institute, Shanghai University, Shanghai, China; 2 Wittgenstein Centre for Demography and Global Human Capital (Univ. Vienna, IIASA, VID/ÖAW), International Institute for Applied Systems Analysis (IIASA), Laxenburg, Austria; 3 School of International Development, University of East Anglia, Norwich, United Kingdom; University of Louvain, BELGIUM

## Abstract

**Background:**

The COVID-19 virus pandemic has caused a significant number of deaths worldwide. If the prevalence of the infection continues to grow, this could impact life expectancy. This paper provides first estimates of the potential direct impact of the COVID-19 pandemic on period life expectancy.

**Methods:**

From the estimates of bias-adjusted age-specific infection fatality rates in Hubei (China) and a range of six prevalence rate assumptions ranging from 1% to 70%, we built a discrete-time microsimulation model that simulates the number of people infected by COVID-19, the number dying from it, and the number of deaths from all causes week by week for a period of one year. We applied our simulation to four broad regions: North America and Europe; Latin America and the Caribbean; Southeastern Asia; and sub-Saharan African. For each region, 100,000 individuals per each 5-year age group are simulated.

**Results:**

At a 10% COVID-19 prevalence rate, the loss in life expectancy at birth is likely above 1 year in North America and Europe and in Latin America and the Caribbean. In Southeastern Asia and sub-Saharan Africa, one year lost in life expectancy corresponds to an infection prevalence of about 15% and 25%, respectively. Given the uncertainty in fatality rates, with a 50% prevalence of COVID-19 infections under 95% prediction intervals, life expectancy would drop by 3 to 9 years in North America and Europe, by 3 to 8 years in Latin America and the Caribbean, by 2 to 7 years in Southeastern Asia, and by 1 to 4 years in sub-Saharan Africa. In all prevalence scenarios, as long as the COVID-19 infection prevalence rate remains below 1 or 2%, COVID-19 would not affect life expectancy in a substantial manner.

**Interpretation:**

In regions with relatively high life expectancy, if the infection prevalence threshold exceeds 1 or 2%, the COVID-19 pandemic will break the secular trend of increasing life expectancy, resulting in a decline in period life expectancy. With life expectancy being a key indicator of human development, mortality increase, especially among the vulnerable subgroups of populations, would set a country back on its path of human development.

## Introduction

The impact of the ongoing coronavirus disease global pandemic which started at the end of 2019 (COVID-19) will last for many years to come. Since the World Health Organization (WHO) confirmed on 22 January 2020 that there was evidence of human-to-human transmission of a novel coronavirus, named 2019-nCOV or COVID-19, outbreaks of COVID-19 have caused a significant number of deaths worldwide. At the time of writing (26 August 2020), according to the Center for Systems Science and Engineering (CSSE) at Johns Hopkins University, the global death toll stands at 819,612, with about one-quarter of coronavirus deaths having occurred in Europe and almost another quarter in the United States (USA) alone [1]. The latest projections from the Institute for Health Metric and Evaluation of the University of Washington projected as many as 309,917 cumulative COVID-19 deaths for the USA, 203,934 deaths for Brazil, 130,387 deaths for Mexico, and 60,897 deaths for the United Kingdom (UK) by December [2]. Currently, the USA, Brazil, Mexico, India, and UK have recorded the highest number of deaths worldwide, but the situation is evolving quickly and countries in other parts of the world are experiencing a sharp and rapid rise in infections and mortality.

SARS-CoV2 virus–severe acute respiratory syndrome coronavirus 2 –is a serious threat to public health and is characterized by some features that differentiate it from most of the epidemics of the last decades. First of all, the transmission rate of COVID-19 is particularly high. With the basic reproduction number (R_0_) ranging from 1.9 to 6.5 [3–5], the virus is highly contagious and can spread very quickly. Part of this high infectivity is due to both asymptomatic and pre-symptomatic cases being able to transmit the virus [6], which is a particularly insidious characteristic in terms of setting up appropriate preventive measures. The high transmissibility, coupled with increased globalization and extensive global mobility, has resulted in outbreaks of COVID-19 in all world regions in a short period of time [7].

The spectrum of illness severity of the virus is also quite unique. Most people manifest no or mild symptoms [8]. This group is estimated to represent 80% of the number of people actually infected [9] although this figure is likely to be underestimated. For the remaining cases, the illness develops into mild to critical bilateral pneumonia, with patients showing symptoms varying from dyspnea to respiratory failure and death. At the moment of writing, infection fatality rates are unknown, as the dimension of the contagion and the number of the asymptomatics has yet to be investigated systematically [10]. Likewise, there is no uniform system for counting deaths across countries [11]. Data coming from highly affected countries like Italy, Spain, the USA, the UK, and China show very different figures from data related to countries that were able to effectively control the contagion such as South Korea. Uncertainty around the infection fatality rates is mainly due to the difficulty of identifying the real incidence and prevalence of COVID infection at any point in time.

From a demographic perspective, the main characteristic of COVID-19 is that the large majority of severe cases involves older populations, especially those aged 70 years and over [12]. According to Italian data, for example, a 40–49-year-old infected by the virus is around 27 times less likely to die than 70–79-year-old [13]. In addition, underlying medical conditions, including hypertension, respiratory system disease, cardiovascular disease, diabetes, and chronic kidney disease are also found to be a risk factor for severe COVID-19 disease [14–16]. Italian data show that only 2% of deceased COVID patients had no comorbidities when they became infected [13].

If mortality from COVID-19 continues to rise, it could have an impact on period life expectancy. Previous epidemics such as the 1918 influenza pandemic and the 2014 Ebola virus outbreak resulted in a drop in life expectancy at birth of as many as 11.8 years and 1.6–5.6 years in the USA and Liberia, respectively [17, 18]. In severely affected countries, an unprecedented surge in mortality from COVID-19 may result in significant years of life lost.

The impact of the COVID-19 epidemic on the life expectancy of a population, however, is not so clear-cut. On the one hand, as the virus kills a disproportionate number of people in the older population, the number of years lost with respect to existing average life expectancy might be smaller than expected. On the other hand, the rapid spread of the virus might cause a high level of excess mortality, as observed in many European countries [19], that is consistently large enough to affect the lifetable of a country or region.

All other things being equal, this paper aims to provide first estimates of the potential direct impact of the COVID-19 pandemic on period life expectancy. We built a discrete-time microsimulation model that simulates the life histories of 100,000 individuals by five-year age groups week by week for a period of one year. To account for a large range of possible outcomes of the pandemic, we built a range of scenarios combining bias-adjusted age-specific infection fatality rates and their 95% credible interval (CrI) estimated for the province of Hubei (China) by Verity et al. [20] and six assumptions of COVID-19 infection prevalence rates. The estimates are carried out for four broad regions that, respectively, have i) a very high life expectancy (North America and Europe, 79.2 years), ii) a high life expectancy (Latin America and the Caribbean, 76.1 years), iii) a medium life expectancy (Southeastern Asia, 73.3 years) and iv) a low life expectancy (sub-Saharan Africa, 62.1 years). Our study shows the impact of lifetables on the outcome of the COVID-19 pandemic, all other things being equal and, accordingly, the outcomes can be transposed to any regions sharing similar lifetables. While this exercise does not serve as a prediction of what will happen to life expectancy in different contexts, it shows what the potential impact on life expectancy would be if the same age-specific infection rates and fatality rates of Hubei province were replicated elsewhere to regions with different population structures.

Note that this exercise does not aim to provide a precise estimate of the years of life lost due to COVID-19, given two important features of the actual pandemic. First, the available evidence regarding the prevalence of COVID-19 infection and second, as a consequence, the infection fatality rates of COVID-19 both remain largely uncertain. Rather than providing an estimate, we offer a range of possibilities based on different scenarios of infection rates. The development of the situation will shed light on the real values of these parameters in different countries. A second reason for focusing on scenarios rather than point estimates is related to a further characteristic of the epidemic, that is, most of the bleakest consequences of COVID-19 have been experienced in specific clusters limited to specific areas of a country.

In the case of Italy, for instance, Bergamo and Lodi, geographically delimited areas in Lombardy in the north of Italy, witnessed massive-scale diffusion of the virus. These areas represent roughly 2% of the entire Italian population. The rest of the Italian provinces, despite being affected, did not see such massive-scale COVID-19 diffusion. Similar patterns are seen in the cases of Wuhan in China, Madrid in Spain, and New York in the USA. The high concentration of infection in one small geographical area is partly due to the successful containment strategies put in place to limit the spread to other parts of these countries [21].

From a demographic perspective, the impact of the virus in terms of life expectancy should therefore be considered rather as cluster-specific. Average life expectancy will probably be strongly affected not so much at a country level but at sub-national clusters, given that severe illnesses and deaths tend to concentrate in one area [22]. Our modeling evidence could thus be applied to specific areas, as well as to countries as a whole, at a given level of prevalence. The results, by providing estimates of life expectancy based on different prevalence scenarios, are adaptable to Bergamo and Madrid, as well as to Italy and Spain.

## Methods

To assess the impact of COVID-19 on life expectancy, we built a discrete-time microsimulation model that simulates the life of individuals week by week for a period of one year (52 weeks).

The model has two main parameters that change across scenarios:

- age-specific probabilities of dying from COVID-19 among the infected population (*f*_*x*_);
- age-specific prevalence rate of COVID-19 infection (*i*_*x*_), distributed over the year following a normal distribution centered on the middle of the year. In the scenarios presented in this paper, the prevalence rates are assumed to be equal among all age groups.

The model also includes additional parameters that are constant across scenarios:

- age-specific probabilities of dying (*q*_*x*_): taken from aggregated lifetables (5-year age groups) (see data section);
- length of illness (z), which is set at 2 weeks, following the general findings that the risk for undetected symptomatic infection after 14 days is very low (1 in 10,000) [23].

From *q*_*x*_, assuming constant intensity, the age-specific probabilities of dying of COVID-19 at time t ($q_{x}^{t}$) are calculated as follows:

$$q_{x}^{t}=1-{\left(1-q_{x}\right)}^{1/(5*52)}$$ Eq 1

*i*_*x*_ are assumed to follow a normal trend over the year centered on week 26 (S.D. = 10). Therefore, the age-specific probabilities of becoming infected at time t ($i_{x}^{t}$) are calculated as follows:

$$i_{x}^{t}=i_{x}*\frac{1}{10\surd \pi }e^{{-0.5(\frac{t-26}{10})}^{2}}$$ Eq 2

We assume that an individual can be infected only once. Therefore, the denominator in the probabilities of infection is adjusted accordingly:

$$t=1,\;i_{x}^{\prime t}=i_{x}^{t}$$

$$t>1,\;i_{x}^{\prime t}=\frac{i_{x}^{t}}{1-\sum_{a=1}^{t-1}i_{x}^{a}}$$ Eq 3

As the probability of dying from COVID-19 infection is only possible during the illness period, *f*_*x*_ is transformed accordingly:

$$f_{x}^{t}=1-{\left(1-f_{x}\right)}^{1/z}$$ Eq 4

For each age group, 100,000 individuals are simulated. Using the Monte Carlo method, the survival of an individual over a year is simulated given three events:

1. First, the death of an individual from all causes of death besides COVID-19 is simulated using $q_{x}^{t}$. An individual who dies is tagged as "death from non-COVID-19 cause" and the simulation stops.
2. If the individual survives and has not been previously infected by COVID-19, the model simulates the infection using $i_{x}^{\prime t}$. In this case, the individual is tagged as infected for week t and t +1. If the individual is not infected, the simulation is repeated from step 1 for week t+1.
3. If the individual is still alive and is tagged as being "infected by COVID-19" (either from week t-1 or week t), the probability of dying from the disease is simulated using $f_{x}^{t}$. If the individual dies, he or she is then tagged as "death from COVID-19" and the simulation stops. If the individual survives, the survival time goes on and the simulation is repeated from step 1 for the next week.

Once the simulation is done, we can estimate age-specific mortality rates with a presence of COVID-19 ($m_{x}^{\prime }$) by dividing the total number of deaths that occur in each age group by the total person-years of exposure in this age group. Using standard lifetable calculations, $m_{x}^{\prime }$ are used to calculate life expectancies adjusted for the impact of COVID-19 $\left(e_{x}^{\prime }\right)$. The loss in life expectancy due to COVID-19 is calculated by subtracting $e_{x}^{\prime }$ from life expectancy not impacted by COVID-19 mortality (*e*_*x*_).

In addition to loss in life expectancy, we also calculated the total fatality rate from COVID-19 by dividing the number of deaths from COVID-19 by the exposed population, weighted by population size of the same age group in the respective world region (*P*_*x*_).

### Data sources

Data for population by age (*P*_*x*_) in 2020 are taken from the United Nations World Population Prospects 2019 [24] (File POP/7-1: Total population (both sexes combined) by five-year age group, region, subregion and country, 1950–2100). Lifetables (*q*_*x*_ and *e*_*x*_) projected in 2020–2025 under the medium variant are also taken from the same source (File MORT/17-1: Abridged lifetable, for both sexes combined, by region, subregion, and country, 1950–2100).

Age-specific fatality rates (*f*_*x*_) and their 95% credible intervals (CrI) are obtained from Verity et al. [20]. Bayesian methods were used on individual case data for patients who died from COVID-19 in Hubei, mainland China, and these provided the first robust estimates of the age-stratified infection fatality ratio adjusted for the different denominator populations, including censoring, demography, and under-ascertainment of cases.

### Scenarios

Scenarios are built combining age-specific fatality rates (*f*_*x*_) with six prevalence-rate (*i*_*x*_) assumptions.

The assumptions for *f*_*x*_ use the estimates from Verity et al. [20] for Hubei province, China, and their lower and upper limit of the 95% credible intervals (CrI). Up to now (April 2020), bias-adjusted age-specific infection fatality rates have not been estimated for other regions. Our calculations thus rely on the assumption that the age-specific infection fatality rates would be the same in all world regions. In other words, our results show how the impact of COVID-19 on life expectancy might be affected by different demographic structures and lifetables, all else being equal. Age-specific fatality rates are presented in Table 1.

**Table 1:** Assumptions on age-specific COVID-19 infection fatality rates (f_x_).

Age	*Central estimate*	*95% Credible interval (CrI)*
**0–9**	0.00%	(0.00%-0.02%)
**10–19**	0.01%	(0.00%-0.05%)
**20–29**	0.03%	(0.01%-0.09%)
**30–39**	0.08%	(0.04%-0.19%)
**40–49**	0.16%	(0.08%-0.32%)
**50–59**	0.60%	(0.34%-1.28%)
**60–69**	1.93%	(1.11%-3.89%)
**70–79**	4.28%	(2.45%-8.44%)
**80+**	7.80%	(3.80%-13.30%)

Source: Verity et al. [20].

Table 1 presents a strong age gradient in fatality from COVID-19, with much higher risk of death for older populations aged 70 and over than for younger adults. Fatality rates are close to 0 for the population aged below 30, and start to increase sharply for those aged 60 and over. They reach 7.8% (95% Credible interval (CrI) 3.80%-13.30%) for the population aged 80 and over. Unfortunately, estimates for this latter age group are not broken down further. In high life-expectancy regions, where there is a much larger number of survivals at an older age than in Hubei, this could lead to a slight underestimation of the fatality rates. Fatality rates in the lower limit of the 95% CrI are approximatively one-half lower, while those in the upper limit are twice higher.

We provide six alternative COVID-19 prevalence rates ranging from 1% to 70%. The 1% assumption would be a scenario in which the propagation of the virus is well-contained, while the 70% prevalence would be a scenario in which the virus is spread widely due to limited public interventions to control transmission. In all scenarios, prevalence rates are assumed to be reached within one year. Prevalence rates are assumed to be uniform across all ages, as, unlike in the fatality rate, there is no clear age pattern in the infection rate [25].

## Results

In Table 2, we first present total fatality rates by regions. Note that these results are based on the assumption that everything else is equal (although we may expect poorer resilience of the health systems in lower-income countries). The results thus show the effect of the age structure on fatality rates.

**Table 2:** Total fatality rate by region, 2020.

Region	f_x_ = central estimate	f_x_ = lower limit *95% CrI*	f_x_ = upper limit *95% CrI*
North America and Europe	1.0%	0.6%	2.0%
Latin America and the Caribbean	0.5%	0.3%	1.1%
Southeastern Asia	0.5%	0.2%	0.9%
Sub-Saharan Africa	0.2%	0.1%	0.4%

The total fatality rate from COVID-19 is 1% in North America and Europe, and ranges from 0.6% under the lower 95% CrI of f_x_ to 2% under the upper one. Total fatality rates are about twice lower in Latin America and the Caribbean and in Southeastern Asia than in North America and Europe and 5 times lower in sub-Saharan Africa, which highlights the role of age structure in vulnerability to mortality risk from COVID-19. Our results are in line with those of Dowd et al. [26], who modeled dramatically more deaths in Italy than in Nigeria under the same prevalence and fatality rate.

**Fig 1:**
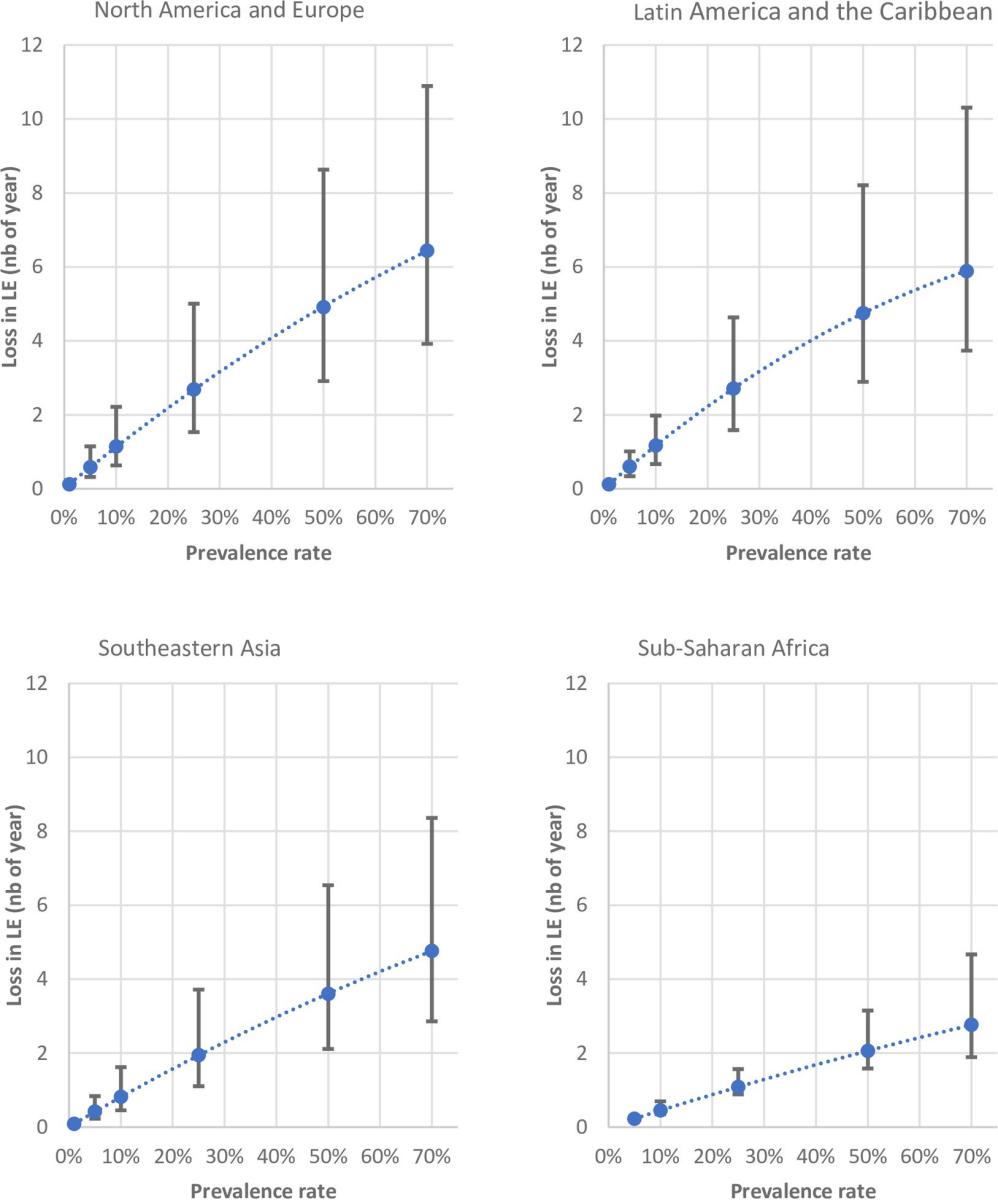
Loss in life expectancy according to different prevalence rates, error bar = 95% credible interval.

In the absence of COVID-19, life expectancies for men and women combined in 2020 were expected to be 79.2 years in North America and Europe, 76.1 years in Latin America and the Caribbean, 73.3 years in Southeastern Asia and 62.1 years in sub-Saharan Africa. Fig 1 shows the loss in life expectancy following different combinations of age-specific fatality rates and prevalence rates assumptions. Detailed results are in S1 Table.

In North America and Europe and in Latin America and the Caribbean, each percentage increase in the prevalence of COVID-19 infection would reduce life expectancy by about 0.1 year. The reduction in life expectancy is slightly steeper when the prevalence is low and becomes flatter when the prevalence gets higher. At an infection prevalence of 10%, a little over one year of life expectancy is lost, and at 50% of infection prevalence, about 5 years are lost.

The impact of COVID-19 on the period life-expectancy would be lower in Southeastern Asia, and even much lower in sub-Saharan Africa. For Southeastern Asia, one year lost in life expectancy corresponds to an infection prevalence of about 15% compared to a prevalence of 25% in sub-Saharan Africa. At 50% infection prevalence, the years of life lost amount to 3.5 years in South Eastern Asia and 2 years in sub-Saharan Africa. In short, loss in life expectancy in sub-Saharan Africa would be half of those in North America and Europe, while years of life lost due to COVID-19 in Southeastern Asia would lie between the other two regions.

Given the uncertainty in the estimate of age-specific infection fatality rates, the number of years lost in life expectancy can fall within the upper and lower 95% CrI of fatality rates. With respect to the upper limit, 11 years of life expectancy are lost at 70% prevalence in North America and Europe, 10 years in Latin America and the Caribbean, 8 years in Southeastern Asia and 5 years in sub-Saharan Africa.

Naturally, the years of life lost in the lower limit are lower, equivalent to 4 years under 70% prevalence of COVID-19 infection in North America and Europe and in Latin America and the Caribbean. In Southeastern Asia and sub-Saharan Africa, the loss would be even smaller, 3 years and 1 year, respectively.

In general, the loss in life expectancy remains low as long as the prevalence does not exceed a certain threshold. Indeed, with a prevalence of infection below 1%, the years of life lost are likely to be smaller than the annual secular increase, which is about 0.2 in high-income countries, and the trend would remain unaffected. However, with an above-20% prevalence of COVID-19 infection, the effect on the secular trend can become sizable. At very high prevalence (70%), the loss in period life expectancy in North America and Europe would range from 4 to 11 years. A clear break in the historical trend might be observed and would be visible when the age pyramids are plotted in the coming few years.

The impact of COVID-19 on life expectancy primarily relies on age-specific mortality rates. Therefore, in all prevalence scenarios, losses are larger in North America and Europe and in Latin America and the Caribbean, where life expectancy is higher, than in Southeastern Asia and sub-Saharan Africa. Indeed, the smaller the number of survivals at older ages in the lifetable, the smaller the impact of COVID-19 on life expectancy. This is because COVID-19 is disproportionately fatal among older age groups.

## Discussion and conclusion

As long as COVID-19 infection prevalence remains low in a region, the pandemic will not substantially affect life expectancy. However, above a certain COVID-19 prevalence threshold, about 2% in high life expectancy regions, the secular increasing trend in life expectancy would be broken by a period drop in life expectancy.

Therefore, a failure to contain the spread of the virus would result in a higher number of deaths [2, 27] and as a consequence lower life expectancy of a sizable magnitude. At merely 2% COVID-19 infection prevalence, the secular increase in life expectancy is likely to be suspended. At 10% prevalence, the loss in life expectancy is likely be above 1 year in high life-expectancy countries. At 50%, it would translate into 3 to 9 years of life lost in high life-expectancy regions, 2 to 7 years in medium life-expectancy regions, and 1 to 4 years in low life-expectancy regions. Life expectancy in North America and Europe could become comparable to what has been observed in Brazil in recent years, while such a loss could temporarily set Latin America and the Caribbean back to the life expectancy they had 20 years ago.

If the true age-specific infection fatality rates are close to the estimated upper limit of the Crl and the infection prevalence reaches 70%, the impact of COVID-19 on life expectancy would be similar to that of the 1918 flu pandemic in the USA [18]. Even in the most advanced countries, this would likely bring life expectancy down to below 70 years, which is equivalent to the life expectancy Western Europe had 60 years ago.

Other studies used excess of mortality to estimate the impact of COVID-19 on life expectancy without relying on any data on infection prevalence. For the state of New York, Heuveline and Tzen [28] calculated a loss of about 2 years in life expectancy from the number of extra deaths in the state between 14 March and 20 May. Using a similar approach, Ghislandi et al. [29] estimated a loss in life expectancy of 4.1 years and 2.6 years for men and women, respectively, in Bergamo (Lombardy), given excess mortality in the period 1 January to 30 April 2020, on the assumption that mortality will go back to the normal trends observed in 2015–2019 for the rest of the year. Serological tests conducted on 9,965 citizens in Bergamo showed that over half of those tested have coronavirus antibodies [30]. In our model, a 50% infection prevalence rate would correspond to 3–9-year loss of life expectancy in high life-expectancy regions. Loss of years of life expectancy in Bergamo would thus be in the lower interval of our estimates.

The results from the studies using excess mortality are not, however, directly comparable to ours. The former is based on true observational data where years of life loss in a specified period when excess mortality was observed are calculated assuming that the excess mortality is directly and indirectly related to COVID-19. Our study, on the other hand, relies on scenarios by simulating the pandemic under different assumptions of mortality and infection rates. Our study provides "what if" scenarios that can give policy-relevant information on what could potentially happen to life expectancy under different levels of prevalence that vary with public health measures to control the spread of COVID-19.

One advantage of our approach, which uses four different lifetables ranging from low to very high life expectancy, is that our estimates can be transposed to any regions or countries that share a similar mortality pattern. Although countries may have managed to contain the prevalence of COVID-19 infection at the national level, it is still possible that the impact of COVID-19 on life expectancy is felt at the sub-national level. For instance, our estimates of high prevalence rate using the lifetable of Latin America and the Caribbean can be used as a proxy for the impact of COVID-19 on human life in severely affected regions in Brazil.

Life expectancy is calculated based on annualized mortality rates. In our simulations, we assumed a normal distribution of infections and subsequent deaths centered in the middle of the year. Our estimates of the impact of COVID-19 on life expectancy thus smooth the peak of infections for which there would be a higher one-off impact than was suggested by annual life expectancy, even under low prevalence scenarios. For instance, using daily death count data, Trias-Llimos et al. [31] calculated a drop in weekly life expectancy ranging from 6.1 to 7.6 years in Spain, for the last week of March and the first week of April, as compared to a 0.8 year loss when translated into an annual life expectancy.

This study provides first assessments of the potential impact of COVID-19 on period life expectancies according to a range of scenarios of prevalence rates over a one-year period. The results should be interpreted in the context that all other things are kept constant, even though this may seem rather unrealistic. The limitation of the study lies in the fact that true case-specific fatality rates are unknown. We rely on the estimates of age-specific infection fatality rates adjusting for biases based on data from Hubei province, China. It is highly likely that the true infection fatality rates in other regions differ from that of Hubei province, given country differentials in policy interventions, health infrastructure, and population behaviors. Indeed, in places where infection rates were very high like Bergamo, Madrid, and New York, overburdened hospitals may result in higher infection fatality rates than places where the healthcare systems have capacity to care for COVID-19 patients. The results from this study represent a preliminary exercise to investigate what the impact of the COVID-19 pandemic on human life.

In fact, mortality rates are not independent of the prevalence of COVID-19 infection. As the prevalence becomes higher, health infrastructures are likely to be overloaded [32] and to be unable to provide care for everyone who needs it, resulting in higher mortality rates both from the virus itself and from other causes. Accordingly, fatality rates from COVID-19 are likely to increase with a higher prevalence rate, and the impact of the virus on life expectancy may be higher. The risk of mortality is also related to the performance of the health system both in terms of access to it and quality of health care services. This problem is likely to be exacerbated in low-income countries where health care systems lack critical-care resources [33]. Furthermore, COVID-19 could have an indirect impact on mortality from other causes, for example as the result of a disruption in routine health care and poorer access to food in low-income countries [34]. On the other hand, the fatality of the virus may decrease as its spreads, given that people who suffer severe symptoms are less likely to contaminate others than those with mild symptoms. It therefore remains to be seen how many years of life will actually be lost following the COVID-19 pandemic.

## Supporting information

S1 Table Number of years lost in life expectancy. (DOCX)
