# Supplementary material for: Assessing the potential impact of COVID-19 on life expectancy
Source: PLoS One. 2020 Sep 17;15(9):e0238678. doi: 10.1371/journal.pone.0238678 (PMC7498023; doi:10.1371/journal.pone.0238678)
Supplement: S1 Table — (DOCX) [file pone.0238678.s001.docx]

**S1 Table. Number of years lost in life expectancy**

| **Age-specific fatality scenario (f_x_)** | **Region** | **Prevalence assumption (i_x_)** | | | | |  |
| --- | --- | --- | --- | --- | --- | --- | --- |
|  |  | *1%* | *5%* | *10%* | *25%* | *50%* | *70%* |
| *Central* | North America and Europe | 0.13 | 0.65 | 1.26 | 2.90 | 5.08 | 6.46 |
|  | Latin America and the Caribbean | 0.12 | 0.60 | 1.17 | 2.71 | 4.75 | 5.89 |
|  | Southeastern Asia | 0.07 | 0.36 | 0.72 | 1.79 | 3.53 | 4.89 |
|  | Sub-Saharan Africa | 0.04 | 0.20 | 0.41 | 1.01 | 2.03 | 2.84 |
| *Lower 95% CrI* | North America and Europe | 0.07 | 0.33 | 0.65 | 1.59 | 3.04 | 4.11 |
|  | Latin America and the Caribbean | 0.07 | 0.34 | 0.67 | 1.59 | 2.89 | 3.74 |
|  | Southeastern Asia | 0.04 | 0.19 | 0.37 | 0.95 | 1.96 | 2.81 |
|  | Sub-Saharan Africa | 0.02 | 0.09 | 0.18 | 0.46 | 0.94 | 1.32 |
| *Upper 95% CrI* | North America and Europe | 0.22 | 1.09 | 2.14 | 4.98 | 8.73 | 10.85 |
|  | Latin America and the Caribbean | 0.21 | 1.01 | 1.98 | 4.63 | 8.21 | 10.31 |
|  | Southeastern Asia | 0.17 | 0.86 | 1.67 | 3.86 | 6.68 | 8.19 |
|  | Sub-Saharan Africa | 0.08 | 0.41 | 0.81 | 2.00 | 3.91 | 5.36 |
